# Digging Deep: Medication Adherence in Chronic Diseases and Its Association With Patient Satisfaction and Stress in an Indian Metropolis

**DOI:** 10.7759/cureus.46493

**Published:** 2023-10-04

**Authors:** Medha H Shah, Amalesh S Honnekeri, Divya A Samat, Priyanshi Shah, Usha V Nayak, Shobha G Kini

**Affiliations:** 1 Pharmacology and Therapeutics, K. J. (Karamshi Jethabhai) Somaiya Medical College, Mumbai, IND; 2 Internal Medicine, Topiwala National Medical College & B. Y. L. Nair Charitable Hospital, Mumbai, IND; 3 Physiology, K. J. (Karamshi Jethabhai) Somaiya Medical College, Mumbai, IND

**Keywords:** physician counselling, indian population, stress, patient satisfaction, chronic diseases, medication adherence

## Abstract

Introduction

Medication adherence is a critical aspect of managing chronic diseases. Poor medication adherence leads to therapeutic failures and increased health costs, and puts patients at potentially life-threatening risks.The impact is felt drastically by patients suffering from chronic diseases. Patient satisfaction is known to be strongly associated with medication adherence. Psychosocial factors such as depression have been proven to negatively affect medication adherence; however, to our best knowledge, the association of stress with adherence remains largely unexplored.

Objectives

The aim of this study is to explore or assess the relationship between medication adherence, patient satisfaction, and stress levels.

Methods

A cross-sectional observational study was conducted within an Indian metropolitan city (Mumbai) among adults diagnosed and treated for at least one chronic disease with a medication regimen spanning over three months. An online questionnaire was designed, incorporating validated scales such as the Adherence to Refills and Medications Scale, Short Assessment of Patient Satisfaction, and Perceived Stress Scale.

Results

In the study, 23.7% of participants (n=300) showed adherence to their prescribed treatment regimen. Adherence exhibited a positive association with age (p=0.009) and educational attainment (p=0.031). Additionally, a significant gender difference emerged, with males (28%) displaying higher adherence rates compared to females (16.7%) (p=0.036). Furthermore, participants reporting lower stress levels exhibited higher adherence (39.5%), while those experiencing moderate-to-high stress levels displayed reduced adherence rates (17-18.8%) (p<0.05). Patient satisfaction was also linked to adherence, as satisfied individuals demonstrated higher adherence levels (29.1%) in contrast to dissatisfied counterparts (15.7%) (p=0.011).

Conclusion

Level of medication adherence is much lower in India as compared to other developed nations. Various demographic factors such as age, sex, and education status influence adherence. Physician counselling plays an important role in adherence, and satisfied patients are far more adherent. Furthermore, a significant negative association was found between stress and adherence.

## Introduction

The World Health Organization defines medication adherence as “the extent to which a person’s behavior - taking medication, following a diet, and/or executing lifestyle changes, corresponds with agreed recommendations from a health care provider.” Poor adherence leads to therapeutic failures and increased health costs, and puts patients at potentially life-threatening risks. The impact is more drastically felt for chronic diseases such as mental disorders, non-communicable diseases, asthma, tuberculosis, and HIV-AIDS [1]. Adherence to long-term therapy for chronic diseases in developed countries averages 50%, whereas in India, it ranges from 16.6% to 24.1% [1-3].

Sociodemographic factors such as age [3-5], sex [4], and marital status [3,4]; medication-related factors such as complexity of the regime [3,4,6], intolerable side effects [4], and cost of drug [5]; and recipient-related factors such as knowledge about the disease [4-6], beliefs about effectiveness of medication [5,7], quality of life [6], lack of social support [5], forgetfulness [3,5], and fear of dependence to the medication [5] have a significant association with adherence.

Patient satisfaction (how the patent rates the quality of care they are receiving) is known to be strongly associated with adherence, and 64.5% of the completely satisfied patients in a study conducted in Rome, Italy, showed good adherence [8].

Perceived stress is defined as feelings or thoughts that people have about how much stress they are under [9]. Psychosocial factors such as depression have been proven to negatively affect adherence [10]; however, to our best knowledge, the association of stress with adherence remained largely unexplored. Therefore, through our study, we aim to shed light on this aspect of adherence.

In the United States, a study demonstrated that men were more likely to be adherent to medication than women [11]. In South India, a study was conducted that assessed various medication-related factors that may be contributing to lack of medication adherence, but there were some statistically insignificant results [12]. Therefore, we attempted to further delve into these factors in order to deepen the already existing body of knowledge on the topic.

This study assessed adherence, examined its relationship with various factors, and established its association with patient satisfaction and perceived stress.

## Materials and methods

Study design

This was a cross-sectional observational study.

Study population

A total of 300 adults residing in Mumbai (an Indian metropolitan city), who have been diagnosed and treated for at least one chronic disease, were recruited to participate in this study.

Inclusion and exclusion criteria

Table 1 represents the various inclusion and exclusion criteria that were essential in identifying and recruiting study participants. As per Table 1, every inclusion criterion had to be met in order for a prospective subject to be deemed eligible as a study participant, while the presence of even one of the exclusion criteria enlisted in Table 1 would disqualify a prospective subject from participating in the study.

**Table 1:** Inclusion and exclusion criteria

Inclusion criteria (every inclusion criterion had to be satisfied in order to be a study participant)	Exclusion criteria (the presence of even one exclusion criterion warranted exclusion from participation in the study as a study subject)
Adults (individuals 18 years or older) living in an Indian metropolitan city	Individuals not diagnosed with and treated for any chronic medical condition
Individuals who have been diagnosed with and treated for at least one chronic disease	Individuals who were not taking regular prescribed medication for more than 3 months
Individuals who have been on regular prescribed medication for more than 3 months	Individuals with an inability to read and understand English, as all the study instruments administered to participants were in English
Individuals who have a working knowledge of English	Individuals not consenting to participate in the study

Data collection method

Participation requests to the eligible candidates (those fulfilling inclusion criteria) were sent out by the student-researcher directly, by word of mouth, or through social media platforms, namely WhatsApp, Instagram, and Facebook. After obtaining their responses, the eligible candidates were sent the following three documents: the survey questionnaire, a participation information sheet, and an informed consent form (see the Appendix for further details on these documents). A scanned copy of the signed Informed Consent Form was obtained from the consenting individuals. The participants were selected based on a simple random sampling method using computer-generated random numbers. Selected participants were informed and thanked about their participation and were requested to fill out the survey questionnaire using electronic forms. Data recorded from the questionnaire was stored on Microsoft Excel spreadsheets and computed for biostatistical analysis.

Study tools

A demographic questionnaire recorded the participant’s age, sex, occupation, education level, and marital status. Next, a questionnaire on the participant's medication details kept a tab of the chronic disease(s) which the participant is suffering from, the total number of medications prescribed, the dosage form/route of the prescribed medications, the daily frequency of the prescribed medications, and the time since the commencement of therapy. The Adherence to Refills and Medications Scale (ARMS) was administered to participants. It is a 12-item 4-point Likert scale used to evaluate self-reported adherence to taking and refilling medications. This instrument has simple wording and hence is appropriate for use among low-literacy patients. The range of possible scores is 12-48, with lower scores indicating better adherence [13]. Another questionnaire studied the factors affecting adherence: medication-related factors, recipient personality-related factors, and other recipient factors were assessed using this questionnaire, which was validated by two subject experts prior to the commencement of the study. The Short Assessment of Patient Satisfaction (SAPS) questionnaire, a 7-item 5-point Likert scale used to assess patient satisfaction with their treatment, was also given to the participants. Scores ranging from 0 to 10 indicated that the respondent was "very dissatisfied," scores from 11 to 18 indicated that the respondent was "dissatisfied," scores from 19 to 26 indicated that the patient was "satisfied," and scores from 27 to 28 indicated that the respondent was "very satisfied" with their treatment [14]. Lastly, the Perceived Stress Scale (PSS-10), a 10-item 5-point Likert scale for measuring the perception of stress, was also included among the study instruments. Scores ranging from 0 to 13 indicated low stress, scores ranging from 14 to 26 indicated moderate stress, and scores ranging from 27 to 40 indicated high perceived stress [9].

Statistical analysis

Qualitative data were represented in the form of frequency and percentage. Among the qualitative data, nominal data included gender, marital status, occupation, and education, while the ordinal data included PSS-10 and SAPS and were represented using mean ± SD and median and interquartile range. The associations between qualitative variables were assessed using the chi-square test, with continuity correction for all 2 x 2 tables, and using Fisher's exact test for all 2 x 2 tables where the chi-square test was not valid due to small counts (excluding association between adherence and SAPS). Quantitative data was represented using mean ± SD and median and interquartile range. Quantitative data included age, number of medications, time since the commencement of therapy, and so on. Quantitative data were compared using an unpaired t-test, and their association was determined using Spearman's rho. MS Excel and PSPP version 1.0.1 were used for statistical analysis. Graphical representation was done in MS Excel 365.

## Results

Table 2 represents the various possible scores on the ARMS and the corresponding proportion of study participants at each level of the scale. A lower ARMS score indicates a higher adherence to medication.

**Table 2:** Distribution of ARMS score total among the study subjects ARMS, Adherence to Refills and Medications Scale

ARMS score	No.	Percentage
12	53	17.7%
13	18	6.0%
14	25	8.3%
15	47	15.7%
16	19	6.3%
17	23	7.7%
18	23	7.7%
19	12	4.0%
20	8	2.7%
21	15	5.0%
22	14	4.7%
23	7	2.3%
24	5	1.7%
25	7	2.3%
26	3	1.0%
27	1	0.3%
28	2	0.7%
29	5	1.7%
30	3	1.0%
31	1	0.3%
32	1	0.3%
33	2	0.7%
34	1	0.3%
36	1	0.3%
38	2	0.7%
42	1	0.3%
45	1	0.3%
Total	300	100.0%

Table 3 represents the total number of participants who were classified as “adherent” to medication, which was 23.7% of the subjects, and 76.3% of the subjects were not adherent to medication.

**Table 3:** Distribution of adherence on ARMS score among the study subjects The percentages of the top two scores were added and classified as adherent and rest as non-adherent. ARMS, Adherence to Refills and Medications Scale

Adherence on ARMS score	No.	Percentage
Adherence	71	23.7%
Non-adherence	229	76.3%
Total	300	100.0%

Table 4 represents the age demographic of the study population and further subclassifies each age category on the basis of their medication adherence. Overall, 85.7% subjects below 19 years of age, 92.0% aged 20 to 29 years, 90.5% aged 30 to 39 years, 85.0% aged 40 to 49 years, 72.2% aged 50 to 59 years, 72.3% aged 60 to 69 years, and 56.3% aged 70 years and above were found to be non-adherent.

**Table 4:** Association between age (years) and adherence on ARMS score among the study subjects As the age increases, the level of adherence increases, and this association is statistically significant, as per the Pearson chi-square test. ARMS, Adherence to Refills and Medications Scale

Age (years)		Adherence on ARMS score		Total
		Adherence	Non-adherence	
Below 19	No.	1	6	7
	%	14.3%	85.7%	100.0%
20 to 29	No.	2	23	25
	%	8.0%	92.0%	100.0%
30 to 39	No.	2	19	21
	%	9.5%	90.5%	100.0%
40 to 49	No.	9	51	60
	%	15.0%	85.0%	100.0%
50 to 59	No.	25	65	90
	%	27.8%	72.2%	100.0%
60 to 69	No.	18	47	65
	%	27.7%	72.3%	100.0%
70 and above	No.	14	18	32
	%	43.8%	56.3%	100.0%
Total	No.	71	229	300
	%	23.7%	76.3%	100.0%

Table 5 represents the sex-based demographic of study participants. Overall, 83.3% male respondents and 72.0% female respondents were found to be non-adherent to medication regimen. This difference in adherence was found to be statistically significant, as per the Pearson chi-square test.

**Table 5:** Association between sex and adherence on ARMS scores among the study subjects The adherence in males (28%) is more than the adherence in females (16.7%), and this association is statistically significant. ARMS, Adherence to Refills and Medications Scale

Sex		Adherence on ARMS score		Total
		Adherence	Non-adherence	
Female	No.	19	95	114
	%	16.7%	83.3%	100.0%
Male	No.	52	134	186
	%	28.0%	72.0%	100.0%
Total	No.	71	229	300
	%	23.7%	76.3%	100.0%

Table 6 represents the PSS scores. Overall, 28.7% study subjects reported experiencing low stress, 60.7% reported experiencing moderate stress, and 10.7% reported experiencing high stress.

**Table 6:** PSS scores among study subjects PSS, Perceived Stress Scale

PSS scores	No.	Percentage
0 to 13 (low stress)	86	28.7%
14 to 26 (moderate stress)	182	60.7%
27 to 40 (high stress)	32	10.7%
Total	300	100.0%

Table 7 depicts the frequency at which the medicines were prescribed to the study subjects, as well as the level of adherence (adherent or not adherent) corresponding to each frequency. As per the Pearson chi-square test, the differences in adherence between the various frequencies at which medicines were prescribed were not found to be statistically significant.

**Table 7:** Association between daily frequency and adherence on ARMS scores among the study subjects The association between adherence and daily frequency is not statistically significant. ARMS, Adherence to Refills and Medications Scale

Daily frequency of the above medication(s)		Adherence on ARMS score		Total
		Adherence	Non-adherence	
Once a day	No.	40	124	164
	%	24.4%	75.6%	100.0%
Twice a day	No.	26	80	106
	%	24.5%	75.5%	100.0%
Thrice a day	No.	5	21	26
	%	19.2%	80.8%	100.0%
Four times a day	No.	0	4	4
	%	0.0%	100.0%	100.0%
Total	No.	71	229	300
	%	23.7%	76.3%	100.0%

Table 8 describes the level of education of the study subjects and shows the adherence to medication at each educational level. It was found that as the level of education increases, the adherence to medication increases. This was found to be statistically significant on analyzing data using the Pearson chi-square test. However, it was also noticed that individuals with a primary level of education were found to have the highest medication adherence among the cohort, which was an exception to the trend that was otherwise seen in this study.

**Table 8:** Association between education level and adherence on ARMS scores among the study subjects As the education level increases, the adherence increases, and this association is statistically significant. For some unknown reason, subjects having a primary level of education have high proportion of adherence. ARMS, Adherence to Refills and Medications Scale

Education level		Adherence on ARMS score		Total
		Adherence	Non-adherence	
Primary level	No.	4	5	9
	%	44.4%	55.6%	100.0%
Secondary level	No.	0	15	15
	%	0.0%	100.0%	100.0%
Higher secondary	No.	11	48	59
	%	18.6%	81.4%	100.0%
Graduate degree	No.	24	88	112
	%	21.4%	78.6%	100.0%
Post-graduate or professional	No.	32	73	105
	%	30.5%	69.5%	100.0%
Total	No.	71	229	300
	%	23.7%	76.3%	100.0%

Table 9 describes the association between various levels of stress as per the PSS and medication adherence, as per the ARMS. It was seen that as the level of perceived stress increases, the adherence to medication decreases. On analysis using the Pearson chi-square test, this association was found to be statistically significant.

**Table 9:** Association between PSS total and adherence among the cases on ARMS score Subjects having low stress have higher levels of adherence (39.5%) and subjects having moderate to high stress have lower levels of adherence (17%), and this association is statistically significant. PSS, Perceived Stress Scale; ARMS, Adherence to Refills and Medications Scale

PSS scores		Adherence on ARMS score		Total
		Adherence	Non-adherence	
0 to 13 (low stress)	No.	34	52	86
	%	39.5%	60.5%	100.0%
14 to 26 (moderate stress)	No.	31	151	182
	%	17.0%	83.0%	100.0%
27 to 40 (high stress)	No.	6	26	32
	%	18.8%	81.3%	100.0%
Total	No.	71	229	300
	%	23.7%	76.3%	100.0%

Table 10 represents the satisfaction of the study participants to their treatment using the SAPS scale.

**Table 10:** Distribution of patient satisfaction based on SAPS scores among the study subjects SAPS, Short Assessment of Patient Satisfaction

SAPS status	No.	Percentage
Very satisfied	11	3.7%
Satisfied	168	56.0%
Dissatisfied	108	36.0%
Very dissatisfied	13	4.3%
Total	300	100.0%

Table 11 shows the correlation between the level of patient satisfaction to treatment and medication adherence. It was found that subjects who were satisfied with their treatment exhibited a higher medication adherence (29.1%) as compared to subjects who were dissatisfied with their treatment, of which only 15.7% reported adherence to medication. On analysis using the Pearson chi-square test, this result was found to be statistically significant.

**Table 11:** Association between satisfaction to treatment and medication adherence Satisfied subjects showed higher adherence (29.1%) as compared to dissatisfied patients (15.7%), and this association is significant. SAPS, Short Assessment of Patient Satisfaction; ARMS, Adherence to Refills and Medications Scale

SAPS status		Adherence on ARMS score		Total
		Adherence	Non-adherence	
Satisfied	No.	52	127	179
	%	29.1%	70.9%	100.0%
Dissatisfied	No.	19	102	121
	%	15.7%	84.3%	100.0%
Total	No.	71	229	300
	%	23.7%	76.3%	100.0%

Table 12 shows the relationship between the patient's occupation and medication adherence. On applying the Pearson chi-square test, this association was not found to be significant.

**Table 12:** Association between patient's occupation and medication adherence. This association was found to not be significant. ARMS, Adherence to Refills and Medications Scale

Occupation		Adherence on ARMS score		Total
		Adherence	Non-adherence	
Service	No.	8	46	54
	%	14.8%	85.2%	100.0%
Business	No.	15	37	52
	%	28.8%	71.2%	100.0%
Medical professional	No.	7	18	25
	%	28.0%	72.0%	100.0%
Professional	No.	6	17	23
	%	26.1%	73.9%	100.0%
Chartered accountant	No.	5	8	13
	%	38.5%	61.5%	100.0%
Self-employed	No.	1	9	10
	%	10.0%	90.0%	100.0%
Legal	No.	1	4	5
	%	20.0%	80.0%	100.0%
Housewife	No.	14	44	58
	%	24.1%	75.9%	100.0%
Retired	No.	11	22	33
	%	33.3%	66.7%	100.0%
Student	No.	3	24	27
	%	11.1%	88.9%	100.0%
Total	No.	71	229	300
	%	23.7%	76.3%	100.0%

Table 13 shows the relationship between patient's marital status and medication adherence. On applying the Pearson chi-square test, this association was not found to be significant. 

**Table 13:** Relationship between patient's marital status and medication adherence. ARMS, Adherence to Refills and Medications Scale

Marital status		Adherence on ARMS score		Total
		Adherence	Non-adherence	
Married	No.	66	194	260
	%	25.4%	74.6%	100.0%
Single	No.	5	35	40
	%	12.5%	87.5%	100.0%
Total	No.	71	229	300
	%	23.7%	76.3%	100.0%

Table 14 shows the relationship between the dosage form/route and medication adherence. This finding was not significant on applying the Pearson chi-square test.

**Table 14:** Relationship between medication dosage form/route and adherence. ARMS, Adherence to Refills and Medications Scale

Dosage form/route of the medication(s)		Adherence on ARMS score		Total
		Adherence	Non-adherence	
Oral	No.	67	194	261
	%	25.7%	74.3%	100.0%
Oral, inhaled	No.	1	21	22
	%	4.5%	95.5%	100.0%
Oral, injection	No.	3	9	12
	%	25.0%	75.0%	100.0%
Inhaled	No.	0	5	5
	%	0.0%	100.0%	100.0%
Total	No.	71	229	300
	%	23.7%	76.3%	100.0%

Table 15 shows various patient beliefs regarding their mediation regimen and associates patient beliefs with medication adherence. This association was not found to be significant on applying the Pearson chi-square test for analysis.

**Table 15:** Relationship between patient's beliefs about medication and medication adherence. ARMS, Adherence to Refills and Medications Scale

Patient's beliefs about medication		Adherence on ARMS score		Total
		Adherence	Non-adherence	
Effective, essential, and safe	No.	41	106	147
	%	27.9%	72.1%	100.0%
Effective, essential but potentially harmful	No.	29	115	144
	%	20.1%	79.9%	100.0%
Ineffective and harmful	No.	1	8	9
	%	11.1%	88.9%	100.0%
Total	No.	71	229	300
	%	23.7%	76.3%	100.0%

Table 16 shows the relationship between perceived medication side effects and medication adherence. On analysis using the Pearson chi-square test, this association was not found to be significant.

**Table 16:** Relationship between perceived medication side effects and medication adherence. ARMS, Adherence to Refills and Medications Scale

Perceived medication side effects		Adherence on ARMS score		Total
		Adherence	Non-adherence	
Yes	No.	16	74	90
	%	17.8%	82.2%	100.0%
No	No.	55	155	210
	%	26.2%	73.8%	100.0%
Total	No.	71	229	300
	%	23.7%	76.3%	100.0%

Table 17 shows the relationship between the patient's feelings of potentially being stigmatized due to the treatment they are taking and medication adherence. This association was found to be significant on analysis with the Pearson chi-square test.

**Table 17:** Relationship between stigma perceived by patients due to treatment and medication adherence. ARMS, Adherence to Refills and Medications Scale

Question to subjects: Do you feel stigmatized by your disease?		Adherence on ARMS score		Total
		Adherence	Non-adherence	
Yes	No.	10	63	73
	%	13.7%	86.3%	100.0%
No	No.	61	166	227
	%	26.9%	73.1%	100.0%
Total	No.	71	229	300
	%	23.7%	76.3%	100.0%

Table 18 shows the relationship between the subject's work schedule and adherence to medication. It was found that this association was significant on analysis using the Pearson chi-square test.

**Table 18:** Relationship between work schedule/daily routine and medication adherence. ARMS, Adherence to Refills and Medications Scale

Question to subject: Does your work schedule/daily routine interfere with your medication regime?		Adherence on ARMS score		Total
		Adherence	Non-adherence	
Yes	No.	6	68	74
	%	8.1%	91.9%	100.0%
No	No.	65	161	226
	%	28.8%	71.2%	100.0%
Total	No.	71	229	300
	%	23.7%	76.3%	100.0%

## Discussion

Assessment of medication adherence

Medication adherence is a crucial factor that determines treatment outcomes in chronic diseases. A cross-sectional study was conducted among 300 subjects suffering from chronic illnesses in an Indian metropolitan city. On the basis of ARMS scores, 23.7% subjects were found to be adherent to their treatment regime. The level of adherence found in our study is similar to other Indian studies. A study conducted among hypertensives in a rural population of Tamil Nadu showed an adherence of 24.1%, whereas another study conducted among diabetic patients showed 16.6% adherence [2,3]. A study conducted in South India among patients with chronic illnesses studied medication-related factors extensively [12]. Another south Indian study conducted among type 2 diabetes mellitus patients in a tertiary care hospital showed 33% adherence [15]. Higher levels of adherence have been seen in studies conducted in other countries like Italy (39.3%), Lebanon (42.6%), Brazil (45.9%), and Scotland (91.4%) [6,16-18].

Factors affecting adherence

Sociodemographic Factors

Age: As seen in Table 4, a significant association was found between age and medication adherence in our study. As age increased, the adherence also increased, and maximum adherence (43.8%) was found in those aged 70 years and above. Similar findings were seen in a previous study conducted in Pakistan, which reported better adherence observed in older people [5]. In another study conducted among hypertensive patients in North India, good adherence was seen in those aged 50 years and above (66.7%) as compared to those below 50 years of age (42.9%) [4].

Sex: As per Table 5, in our study, males (28%) were found to be more adherent than females (16.7%), and this association was statistically significant. A study conducted in the United States showed similar findings. It was believed that women, who are frequently the primary caregivers, spend less time and energy taking care of themselves. Patient education targeted toward the female population could prove useful [11]. However, a study conducted in rural Vietnam showed no significant association of sex with adherence [19]. Good adherence was seen in males (59.5%) than females (54%) in a study conducted among hypertensive patients in North India, but this association has not proved to be significant [4].

Education level: As seen in Table 8, a significant association was found between education level and adherence. Subjects with higher education level showed better adherence, with high levels of adherence seen in post-graduates (30.5%). Contrary to findings from other education levels, the adherence in primary educated subjects was high (44.4%). This is probably because 77.7% of these subjects are above 70 years of age and 77.7% are satisfied with their treatment. Hence, age and patient satisfaction could be responsible for these deviated findings. Our study findings are consistent with a study conducted among hypertensive patients in Poland, where high adherence to treatment recommendations was observed in patients who had a higher education level [20].

Marital status: As shown in Table 13, in our study, married subjects showed higher adherence (25.4%) compared to unmarried subjects (12.5%); however, this association was not statistically significant. These findings are similar to a north Indian study, which revealed that married patients (60.4%) had better adherence as compared to unmarried patients (43.9%), but this association did not prove to be significant [4].

Occupation: As indicated in Table 12, there is no significant association between occupation and adherence. Further investigation into individual cases is required to evaluate the effect of demographic factors on adherence.

Medication-Related Factors

Complexity of regime: As seen in Table 7 and Table 14, there was no significant association between adherence and dosage form/route and daily frequency of medication consumption. This is similar to findings from a study conducted among diabetics and hypertensives in south India, where no significant association was found between adherence and frequency of medications per day, presence of other comorbidities, and duration of illness [12].

Intolerable side effects: As shown in Table 16, subjects experiencing intolerable side effects of the drug showed lower adherence (17.8%), but this association was not significant. These findings were dissimilar to an Ethiopian study that showed that patients who experience adverse drug effects from their medication are more likely to be non‐adherent and have a poor treatment outcome [21]. In a study to assess compliance with alendronate treatment for osteoporosis, 71% of women who characterized the side effects they experienced as “very” or “extremely” bothersome discontinued the therapy [22]. There is no significant association between non-availability of medication in the market and adherence.

Patient-Related Factors

Knowledge about the disease: It is important that patients obtain correct information from a reliable source. Hence, physician counselling is primary in improving patient knowledge and adherence. This was emphasized in a study conducted among Lebanese patients that showed that physician counselling had a direct effect on patient’s knowledge, which had been found to decrease the risk of non-adherence by twofold [6]. In a study in which patients received educational materials, referral for bone densitometry, and physician consultation, 67% were compliant with treatment after six months [23].

Beliefs about medication: As seen in Table 15, subjects who believed that their medications were effective, essential, and safe showed higher adherence (27.9%) than subjects who thought that their medication was ineffective and harmful (11.1%), but this has proved to be insignificant in our study. A cross-sectional study conducted in the United Kingdom on 324 patients revealed that 89% patients believed that their prescribed medication was necessary for maintaining health, and over a third had strong concerns about the dangers of dependence or long-term effects. Those who believed medications to be necessary reported higher adherence and those with higher concerns correlated with lower adherence [7]. A Pakistani study showed that 82% and 80% of the adherent subjects understood the need for medication and the effectiveness of the medication, respectively [5].

Social support: A study conducted in Pakistan among 432 hypertensives revealed that availability of social support resulted in high level of adherence (82%) [5]. In India, the presence of joint families provides social support, and this might be the cause of good adherence in the older age group. It is commonly seen that the younger members take complete responsibility of the older individuals in their family. However, in our study, there is no significant association between the presence of social support and adherence.

Stigma: As per Table 17, subjects who felt stigmatized by their disease were 13.7% adherent, whereas subjects who did not face stigma were 26.9% adherent. Previous studies have shown that stigma is a major factor for non-adherence. Data examined from 332 patients from six basic psychiatric diagnostic categories showed a statistically significant negative correlation between self-stigma and adherence to treatment in all diagnostic groups [24]. A cross-sectional study conducted among 180 HIV patients in Birmingham showed that higher levels of HIV-related internalized stigma and concerns about being seen by others while taking HIV medication were associated with poor medication adherence [25]. A thorough understanding of interpersonal factors affecting adherence is crucial to achieve long-term positive health outcomes.

Work schedule and daily routine: Table 18 revealed that subjects whose work schedule was affected by their medication regime were far less adherent (8.1%) than others (28.8%). Our findings are similar to a study conducted on youths taking oral diabetes medications in the United States. The study showed that “forgetting” due to disruptions in daily schedule and routine was the most frequent explanation given for missed doses of medication [26]. Travel outside the city, following alternative branches of medicine, and religious beliefs were proved to be not associated with adherence.

Patient satisfaction and adherence

Patient satisfaction was measured using SAPS scores which revealed that satisfied patients had higher levels of adherence (29.1%) as compared to dissatisfied patients (15.7%), and this association was statistically significant (Tables 10, 11). Our study findings are consistent with a longitudinal study in Italy, which showed that 64.5% of the completely satisfied patients showed good adherence [8].

Stress and adherence

PSS-10 scores were used to assess the level of stress in the study subjects. A significant negative association was found between stress and adherence (Table 9). Subjects having low stress had higher levels of adherence (39.5%), and subjects having moderate-to-high stress had lower levels of adherence (17-18.8%). On binary logistic regression analysis, stress was found to be a statistically significant predictor of adherence status among other independent variables such as patient satisfaction, complexity of regime, total number of medications prescribes, knowledge about the disease, intolerable side effects, and time since the commencement of therapy. Our study is among the few studies that explore the relationship between stress and medication adherence, and further corroborative evidence is required.

Limitations of this study

This study relied on self-reported answers and, therefore, could potentially contain some element of recall bias. Additionally, this study made use of extensive scales and questionnaires, and the volume of questions that participants were required to answer could act as a deterrent while participating in the study.

## Conclusions

This study helped us better understand factors that influence medication adherence and showed us the importance of accounting for the patient's choice and lifestyle while designing a therapeutic regimen. We learnt that the patient's demography and educational background, beliefs regarding their therapy, fear of social stigma, and daily schedule significantly hindered adherence, as did the medication dosage form and route. Liaising with the patient to come up with a calculated timetable and daily routine for consuming medication, effectively counselling the patient regarding their medication and assuring them that their treatment is confidential, regularly checking in on them to assess if they are experiencing any intolerable adverse effects, accounting for their personal beliefs, and providing them with a list of alternatives to their therapy along with all the relevant information, in a manner that is clearly understood by them, are paramount in achieving better medication adherence.

## Appendices

Study Questionnaires:

Subject Demography

1.       Age (in years)

a.       18 - 29

b.       30 - 39

c.       40 - 49

d.       50 - 59

e.       60 - 69

f.        70 and above

2.       Sex

a.       Male

b.       Female

c.       Other

3.       Occupation

4.       Educational Level

a.       Primary school certificate (any literacy level below grade VIII)

b.       Middle school certificate (cleared grade VIII examination)

c.       High school certificate (cleared grade X examination)

d.       Higher secondary certificate (cleared grade XII examination)

e.       Graduate degree

f.        Post-graduate or professional

5.       Marital status

a.       Married

b.       Single

Subject Medication Details

1.     Ailment(s)

2.     Total number of medications prescribed for the above ailment(s)

3.     Dosage form/route of above medication(s)

i)      Oral

ii)    Inhaled

iii)  Injection

iv)   Other

4.    Daily frequency of above medication(s)

i)     Once a day

ii)    Twice a day

iii)   Thrice a day

iv)   Four times a day

5.    Time since the commencement of therapy

Adherence to Refills and Medications Scale (ARMS)

It is common for people to miss taking their medicine from time to time, or to take it differently than prescribed. I would like to ask you about how you actually take your medicines. There are no right or wrong answers. For each question, please answer “none of the time,” “some of the time,” “most of the time,” or “all of the time.”

None - 1; Some - 2; Most - 3; All - 4

1.     How often do you forget to take your medicine? 1 2 3 4

2.     How often do you decide not to take your medicine? 1 2 3 4

3.     How often do you forget to get prescriptions filled? 1 2 3 4

4.     How often do you run out of medicine? 1 2 3 4

5.     How often do you skip a dose of your medicine before you go to the doctor? 1 2 3 4

6.     How often do you miss taking your medicine when you feel better? 1 2 3 4

7.     How often do you miss taking your medicine when you feel sick? 1 2 3 4

8.     How often do you miss taking your medicine when you are careless? 1 2 3 4

9.     How often do you change the dose of your medicines to suit your needs (like when you take more or less pills than you’re supposed to)? 1 2 3 4

10.  How often do you forget to take your medicine when you are supposed to take it more than once a day? 1 2 3 4

11.  How often do you put off refilling your medicines because they cost too much money? 1 2 3 4

12.  How often do you plan ahead and refill your medicines before they run out? 1 2 3 4

Factors Affecting Adherence

Medication-related factors

1.     Do you think that the duration of your treatment is too long? Yes/No

2.     Do you feel that your regime is very complex? (excluding too many tablets, too frequent dosing) Yes/No

3.     Have you faced intolerable side effects of the medication? Yes/No

4.     Does non-availability of the medication in the market pose a challenge to you? Yes/No

5.     Did you follow the physician’s instructions while taking your medication? Yes/No

Recipient-related factors

1.     Have you tried to obtain knowledge about your disease from any of the following sources?

a.     Internet

b.     Books

c.     Newspapers/magazines

d.     Peers and relatives

e.     Other

f.      Not tried obtaining knowledge about the disease

2.     What are your beliefs about medication?

a.     Effective, essential, and safe

b.     Effective, essential but potentially harmful

c.     Ineffective and harmful

3.     Do you think that not taking your medicines regularly would worsen your ailment? Yes/No

4.     Has your medication affected the following?

a.     Sleep Yes/No

b.     Appetite Yes/No

c.     Bowel habits Yes/No

d.     Sexual activity Yes/No

5.     Is there someone to remind you to take your medicines if you forget to take them? Yes/No

6.     Do you think that your medication will lead to dependence/addiction? Yes/No

7.     Do you feel stigmatized by your disease? Yes/No

Other recipient factors

1.     Does your work schedule/daily routine interfere with your medication regime? Yes/No

2.     Does your travel outside the city interfere with your medication regime? Yes/No

3.     Do you take any Ayurveda/Homeopathy/Naturopathy/Unani/Siddha medicines as a substitute (partially or wholly) for your current medication treatment? Yes/No

4.     Do your religious beliefs interfere with your treatment regime? Yes/No

Perceived Stress Scale

The questions in this scale ask you about your feelings and thoughts during the last month. In each case, you will be asked to indicate by circling how often you felt or thought a certain way.

0 = Never; 1 = Almost Never; 2 = Sometimes; 3 = Fairly Often; 4 = Very Often

1.     In the last month, how often have you been upset because of something that happened unexpectedly? 0 1 2 3 4

2.     In the last month, how often have you felt that you were unable to control the important things in your life? 0 1 2 3 4

3.      In the last month, how often have you felt nervous and “stressed”? 0 1 2 3 4

4.     In the last month, how often have you felt confident about your ability to handle your personal problems? 0 1 2 3 4

5.     In the last month, how often have you felt that things were going your way? 0 1 2 3 4

6.     In the last month, how often have you found that you could not cope with all the things that you had to do? 0 1 2 3 4

7.     In the last month, how often have you been able to control irritations in your life? 0 1 2 3 4

8.     In the last month, how often have you felt that you were on top of things? 0 1 2 3 4

9.     In the last month, how often have you been angered because of things that were outside of your control? 0 1 2 3 4

10.  In the last month, how often have you felt difficulties were piling up so high that you could not overcome them? 0 1 2 3 4

Short Assessment of Patient Satisfaction (SAPS)

1.     How satisfied are you with the effect of your treatment/care?

a. Very satisfied (0)

b. Satisfied (1)

c. Neither satisfied nor dissatisfied (2)

d. Dissatisfied (3)

e. Very dissatisfied (4)

2.     How satisfied are you with the explanations the doctor/other health professional has given you about the results of your treatment/care?

a. Very dissatisfied (0)

b. Dissatisfied (1)

c. Neither satisfied nor dissatisfied (2)

d. Satisfied (3)

e. Very satisfied (4)

3.     The doctor/other health professional was very careful to check everything when examining you.

a. Strongly agree (0)

b. Agree (1)

c. Not sure (2)

e. Disagree (3)

f. Strongly disagree (4)

4.     How satisfied were you with the choices you had in decisions affecting your health care?

a. Very dissatisfied (0)

b. Dissatisfied (1)

c. Neither satisfied nor dissatisfied (2)

d. Satisfied (3)

e. every satisfied (4)

5.     How much of the time did you feel respected by the doctor/other health professional?

a. All of the time (0)

b. Most of the time (1)

c. About half the time (2)

d. Some of the time (3)

e. None of the time (4)

6.     The time you had with the doctor/other health professional was too short.

a. Strongly agree (0)

b. Agree (1)

c. Not sure (2)

d. Disagree (3)

e. Strongly disagree (4)

7.     Are you satisfied with the care you received in the hospital/clinic?

a. Very satisfied (0)

b. Satisfied (1)

c. Neither satisfied nor dissatisfied (2)

d. Dissatisfied (3)

e. Very dissatisfied (4)
